# Early experience with the ARTISENTIAL^®^ articulated instruments in laparoscopic low anterior resection with TME

**DOI:** 10.1007/s10151-022-02588-y

**Published:** 2022-02-10

**Authors:** I. Darwich, M. Abuassi, R. Aliyev, M. Scheidt, M. A. Alkadri, A. Hees, S. Demirel-Darwich, M. Chand, F. Willeke

**Affiliations:** 1 Department of Surgery, St. Marienkrankenhaus Siegen, Kampenstr. 51, 57072 Siegen, Germany; 2 Wellcome EPSRC Centre for Interventional and Surgical Sciences (WEISS), University College London, 43-45 Foley Street, London, W1W 7JN UK

**Keywords:** Articulation, Laparoscopy, Low anterior resection, Colorectal surgery, ARTISENTIAL

## Abstract

**Background:**

The notion of articulation in surgery has been largely synonymous with robotics. The ARTISENTIAL^®^ instruments aim at bringing advanced articulation to laparoscopy to overcome challenges in narrow anatomical spaces. In this paper, we present first single-center results of a series of low anterior resections, performed with ARTISENTIAL^®^.

**Methods:**

Between September 2020 and August 2021, at the Department of Surgery, St. Marienkrankenhaus Siegen, Siegen, Germany, patients with cancer of the mid- and low rectum were prospectively enrolled in a pilot feasibility study to evaluate the ARTISENTIAL^®^ articulated instruments in performing a laparoscopic low anterior resection. Perioperative and short-term postoperative data were analyzed.

**Results:**

Seventeen patients (10 males/7 females) were enrolled in this study. The patients had a median age of 66 years (range 47–80 years) and a median body mass index of 28 kg/m^2^ (range 23–33 kg/m^2^). The median time to rectal transection was 155 min (range 118–280 min) and the median total operative time was 276 min (range 192–458 min). The median estimated blood loss was 30 ml (range 5–70 ml) and there were no conversions to laparotomy. The median number of harvested lymph nodes was 15 (range 12–28). Total mesorectal excision (TME) quality was ‘good’ in all patients with no cases of circumferential resection margin involvement (R0 = 100%). The median length of stay was 9 days (range 7–14 days). There were no anastomotic leaks and the overall complication rate was 17.6%. There was one unrelated readmission with no mortality.

**Conclusions:**

Low anterior resection with ARTISENTIAL^®^ is feasible and safe. All patients had a successful TME procedure with a good oncological outcome. We will now seek to evaluate the benefits of ARTISENTIAL^®^ in comparison with standard laparoscopic instruments through a larger study.

**Supplementary Information:**

The online version contains supplementary material available at 10.1007/s10151-022-02588-y.

## Introduction

Laparoscopy is considered the gold standard in colon surgery but there remains some skepticism of its role in rectal cancer due to the increased technical difficulty of operating within the narrow pelvis leading to suboptimal results [1, 2]. Advanced instrumentation is a potential solution to overcome the challenges of low anterior resection.

Robotic surgical systems are well known for features such as three-dimensional (3D) and high-definition (HD) vision, digital interactive displays, real-time fluorescence imaging technology, ergonomic telemanipulation, a stable camera command, tremor filtration and motion scaling [3, 4]. Yet, articulation stands out as one of the most striking features of robotic surgery [5, 6]. The increased accessibility and manipulation of tissue provided by an articulating instrument has been suggested to enhance dexterity and augment surgical precision [7]. Furthermore, some non-randomized studies that compared robotic surgery to standard laparoscopy reported improved clinical outcome in favor of robotics [8, 9]. This was mainly observed in terms of the conversion-to-open rate, preservation of autonomic nerves as well as estimated blood loss (EBL) [10, 11]. However, randomized trials failed to confirm these reports while systematic reviews invariably revealed prolonged operating times [12, 13]. Taking this current level of evidence into consideration, the high acquisition, maintenance and running costs of a surgical robotic system are consistent hurdles still limiting the wide spread use of this technology [14].

Previous efforts to introduce articulation to standard laparoscopic instruments have not been successful due to a multitude of factors including unmatured and non-ergonomic design as well as a missing consensus on the optimal technical execution to meet surgeons’ acceptance [15].

In 2019, LIVSMED Inc. (Seongnam, Republic of Korea) introduced ARTISENTIAL^®^, a complete suite of single-use articulated hand-held laparoscopic instruments, featuring a multi-degree-of-freedom level of dexterity and a 360-degree wristed capability of the end effector, similar to that known from surgical robotic systems [16]. The instruments are available with both types of diathermy, bipolar and monopolar. Clinical use of ARTISENTIAL^®^ in multiple surgical disciplines has been reported in several publications recently [17–20].

After acquiring ARTISENTIAL^®^ early in 2020, our surgical department initiated a dry lab training for its medical staff. Soon afterwards, our group conducted a study which confirmed at least a good understanding and command of the ARTISENTIAL^®^ instruments by our surgeons [21]. Since then, more than 40 surgical procedures, including colorectal resections for benign and oncologic indications, mesh rectopexies and hernia repair, were performed with ARTISENTIAL^®^ at our institute. Two case reports with video vignettes reporting the clinical use of ARTISENTIAL^®^ have already been published by our group [22, 23].

The purpose of this single-center study was to evaluate the feasibility and safety of low anterior rectal resection performed with ARTISENTIAL^®^.

### Materials and methods

#### Study population and general information

Between September 2020 and August 2021, at the Department of Surgery, St. Marienkrankenhaus Siegen, Siegen, Germany, consecutive patients scheduled for laparoscopic low anterior resection (LAR) for cancer of the mid- and low rectum (less than 12 cm from the anal verge) were enrolled in a prospectively collected database. A planned open procedure constituted the only exclusion criterium; however, no open procedures were performed during the study period. The patients were part of a prospective cohort study that was approved by the institutional review and ethics board of the Mannheim medical university (protocol no. 2019-417M-§ 23b MPG). Written informed consent for low anterior resection (Perimed Rektumresektion^®^), ARTISENTIAL^®^ assisted surgery, pseudonymized data collection, scientific analysis and publication of scientific material, was obtained from all patients. Two board-certified surgeons, specialized in colorectal surgery, performed all procedures (ID and FW).

Neoadjuvant long-course chemoradiation (nCRT) or short-course radiation therapy (nRT) had been administered to some of the patients in the study according to the decision of the institutional multidisciplinary team. All patients in this study received mechanical bowel preparation and oral antibiotics 1 day before surgery as well as a perioperative intravenous single-dose antibiotic prophylaxis.

All procedures were recorded on video. Time to rectal transection, total operating time, estimated blood loss (EBL) and the number of trocars used were documented for every procedure. All intraoperative complications (bowel, bladder, major vascular or ureter injury) were to be recorded. If iatrogenic injury were caused by ARTISENTIAL^®^, it would be defined as such. Instrument failure or malfunction was recorded and described in details. Two forms of conversion were defined: conversion to laparotomy (CL) and conversion to using standard straight laparoscopic devices (CSL). The Clavien–Dindo classification was used to grade the 30-day postoperative morbidity according to prospectively collected data on complications. Symptomatic grade B and grade C anastomotic leaks were to be recorded according to the classification of the International Study Groups of Rectal Cancer (ISREC) [24, 25].

#### *ARTISENTIAL*^*®*^

**Fig. 1:**
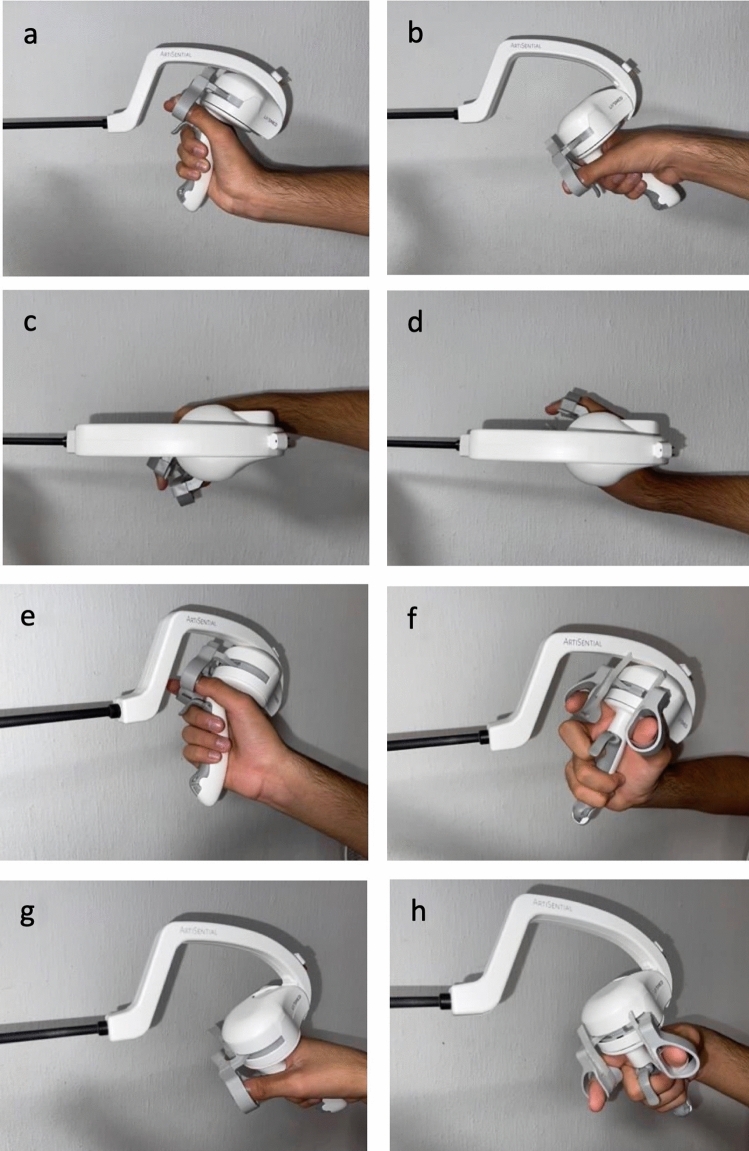
The range of motion of the ARTISENTIAL^®^ handle grip: **a** up, **b** down, **c** left, **d** right, **e** up-right, **f** up-left, **g** down-right, **h** down-left

**Fig. 2:**
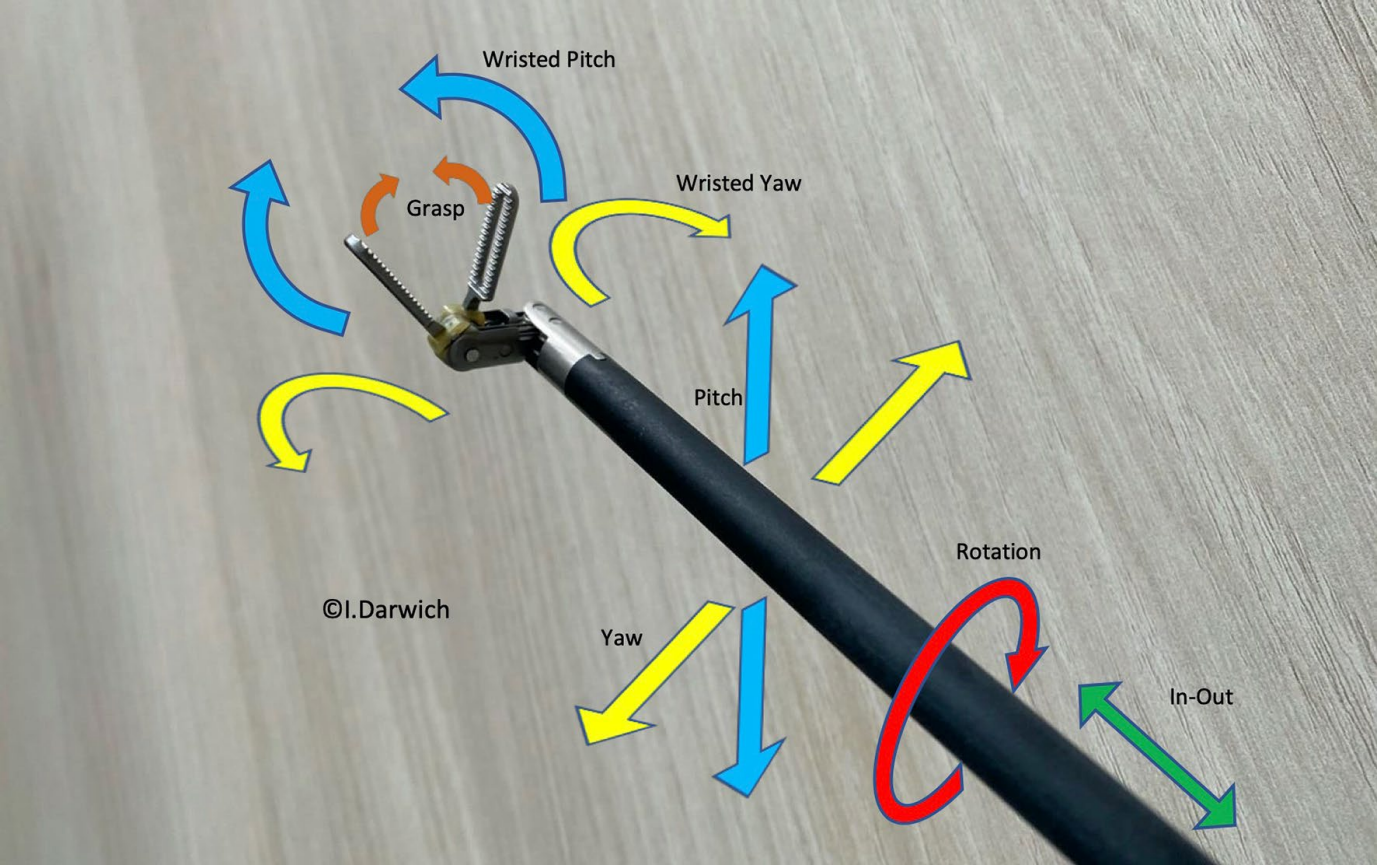
The range of motion of the ARTISENTIAL^®^ end effector

**Fig. 3:**
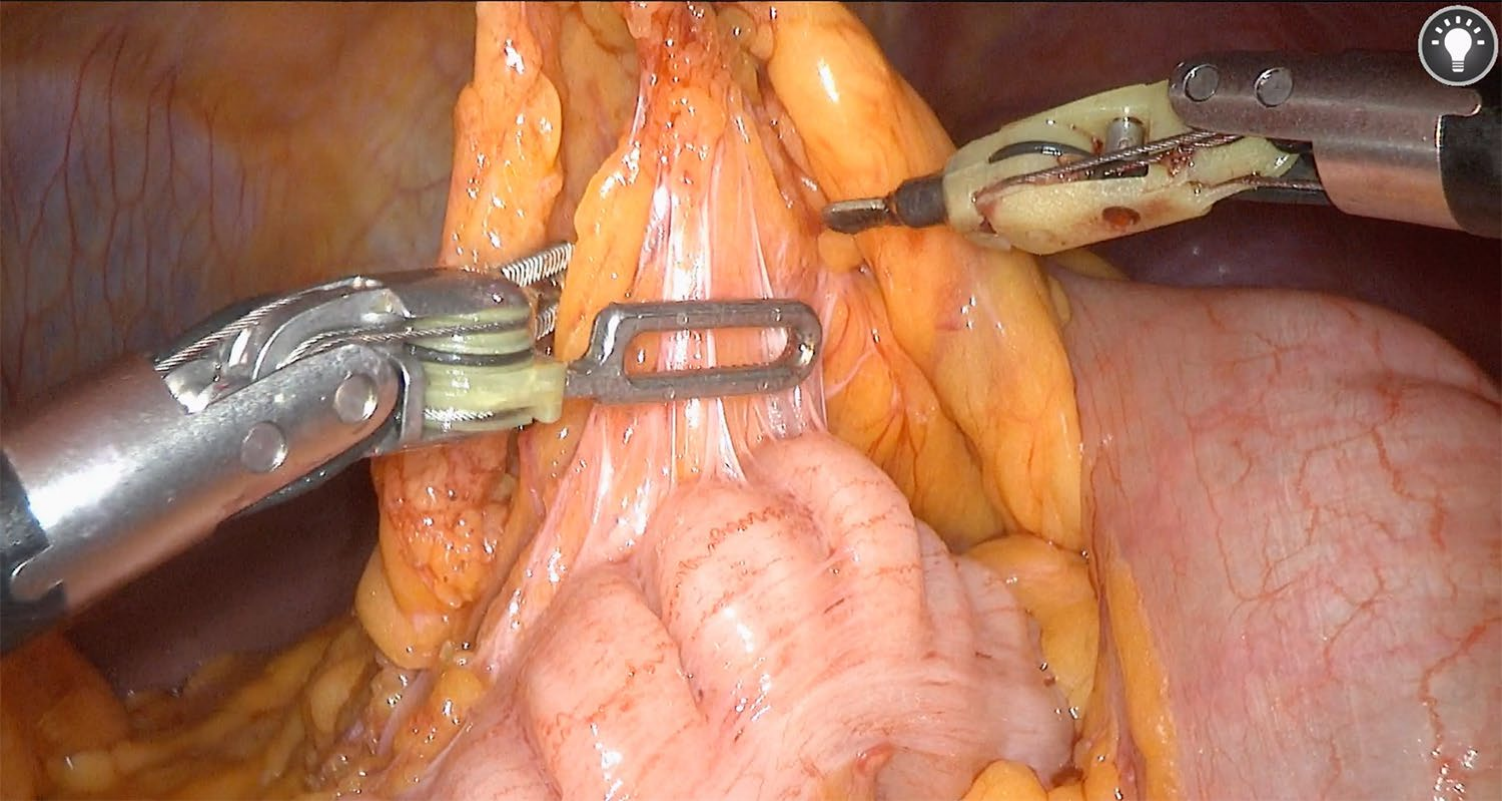
An intraoperative view of the ARTISENTIAL^®^ monopolar spatula and the bipolar forceps

ARTISENTIAL^®^ comprises a complete line-up of purely mechanical, hand-held and single-use laparoscopic instruments that are able to exactly transfer the motion of the user’s thumb and index fingers to the end effector. This is made possible by a kinematic chain of pulleys, cables and joints that connect the handle grip of the instrument to a two-joint end effector along an 8 mm diameter shaft. The handle grip itself is constructed in such a way so as to allow for movement of the hand in the horizontal and in the vertical plane (Fig. 1). Adding bidirectional rotation of the hand along the axis of the forearm, a 360° hemispherical space of motion becomes available for the end effector (Fig. 2). This basically eliminates the fulcrum effect principle, known from standard laparoscopy [21]. All procedures in this study were performed with the ARTISENTIAL^®^ monopolar spatula and the ARTISENTIAL^®^ bipolar forceps (Fig. 3). ARTISENTIAL^®^ is Food and Drug Administration (FDA) approved and has a Conformité Européenne (CE) mark.

#### Surgical technique

**Fig. 4:**
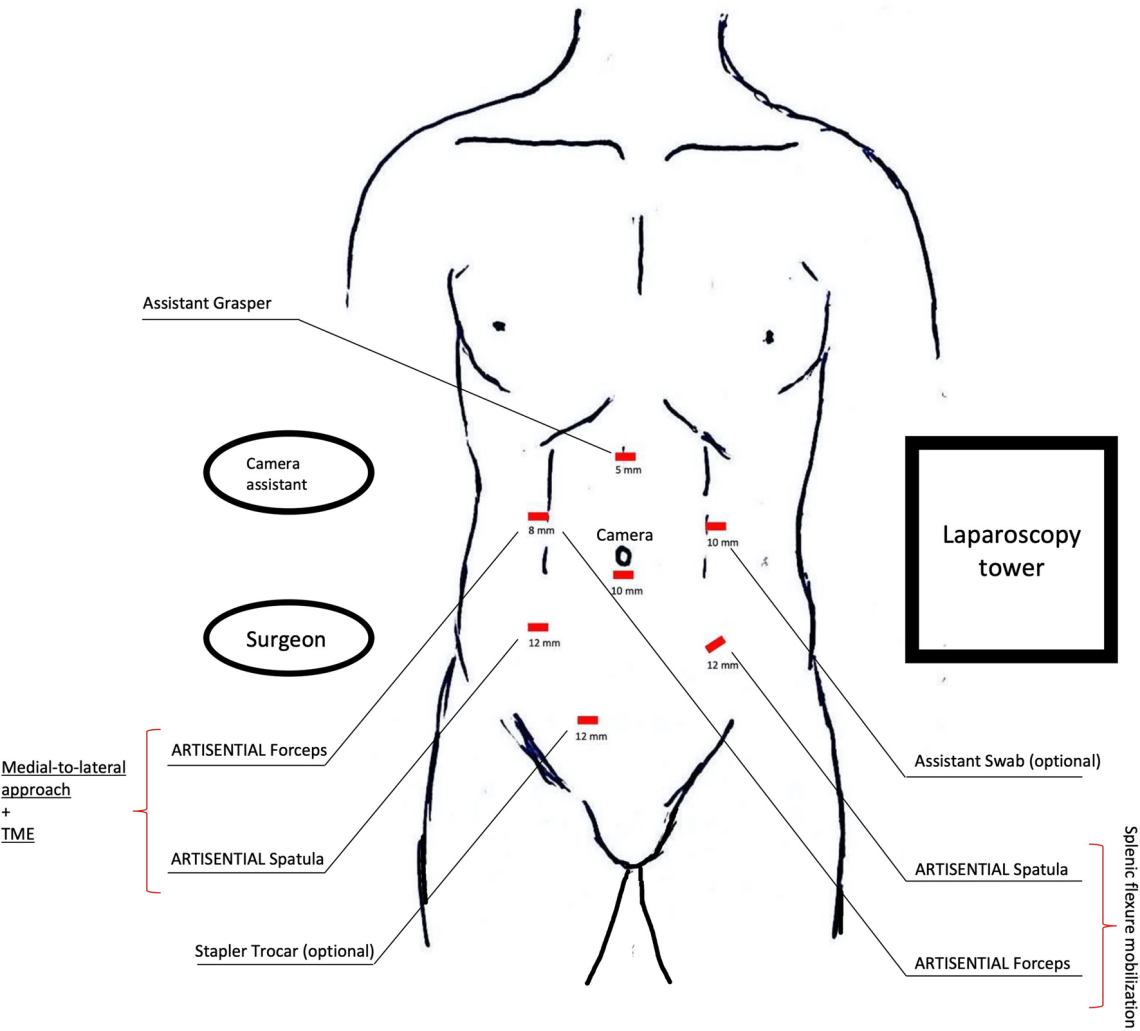
Port placement, trocar sizes and intraoperative setting. The ports in the left hypochondriac and right inguinal region are auxiliary

A standard modified lithotomy position was used. The patient was secured in a surgical bean bag positioner. The upper and lower extremities were sufficiently padded. Port placement, port number, trocar sizes and the intraoperative setup are illustrated in Fig. 4. The camera assistant provided traction using a grasper through the 5 mm trocar in the epigastric region. The 10-mm-sized assistant trocar in the left hypochondriac region was optional and was placed according to the surgeon’s preference in case a swab was needed to lift up the pelvic anterior peritoneal reflection (Fig. 4). The 12-mm right-sided suprapubic trocar was optionally placed to enable stapler-assisted rectal transection in a very narrow pelvis. Trocar positioning was identical to the standard adopted for laparoscopic LAR in our department.

Laparoscopic LAR with total mesorectal excision (TME) was performed according to the principles described by Heald et al. and Enker et al. [26, 27]. A medial-to-lateral approach was performed. The inferior mesenteric vein was divided at the level of the Treitz ligament. A high ligation of the inferior mesenteric artery (IMA) followed while the left ureter and nerves of the superior hypogastric plexus were identified and preserved. After entering the lesser sac, lateral dissection along the white line of Toldt continued so as to connect the planes and totally mobilize the sigmoid and descending colon. The splenic flexure was taken down and dissection along the mesocolic plane continued until the origin of the middle colic artery was identified. TME and rectal transection followed. The time to rectal transection was specifically recorded since all steps until this point were carried out with ARTISENTIAL^®^ whereas the remaining time included steps that did not necessarily involve ARTISENTIAL^®^, like exteriorizing the specimen, extracorporeal skeletonizing of the bowel and fashioning a defunctioning loop ileostomy. The bowel was exteriorized by extending the incision in the left lumbar region. It was then skeletonized extracorporeally at the determined level of proximal transection. Bowel perfusion was assessed with Indocyanine green (ICG)-enhanced fluorescence angiography before transection was performed [28–30]. The anvil of a circular stapling device was then introduced into the colon via an antimesenteric longitudinal colotomy, if a side-to-end double-stapled anastomosis was to be fashioned. If a coloanal anastomosis was planned, the specimen was exteriorized trans-anally. Intraoperative anastomotic integrity was checked with a gas leak test and flexible rectoscopy in all patients.

All patients received a defunctioning loop ileostomy unless a Hartmann procedure was performed.

Anastomotic integrity was examined on follow-up in all patients prior to reversal of the loop ileostomy, using a water-soluble contrast enema, a rectal digital examination and a rectoscopy.

#### Learning curve

Total operative times and times to transection were plotted against a chronological order of the performed cases. Furthermore, a cumulative sum technique (CUSUM) was utilized to try to quantify the learning curve in terms of the time to transection [31–33]. Recursive calculation was performed using the following formula: CUSUM_TT*n*_ = (*X*_TT*n*_ − *M*_TT_) + CUSUM_TT*n* − 1_: (TT: abbr. transection time, *n*: case number, *X*: transection time in minutes, *M*: mean of transection times in minutes). Time to transection was chosen for this analysis instead of total operative time since, as mentioned before, all steps until this point were performed solely with ARTISENTIAL^®^.

#### Statistical analysis

The Kruskal–Wallis test and Fisher exact test were used to check for significance of associations. Associations between operative times and body mass index (BMI) as well as between gender and BMI were analyzed with the Kruskal–Wallis test. Correlations between the number of trocars used and the BMI as well as the between the number of trocars used and gender were analyzed with the Fisher exact test. Data analysis was performed using Microsoft^®^ Excel for Mac (Version 16.55, 2019, Microsoft Corp, Redmond, WA, USA). CUSUM learning curve phases were compared by analysis of variance (ANOVA). Statistical significance was set at *p* < 0.05.

### Results

Seventeen patients (10 males) were prospectively enrolled in this study. The median age of the patients was 66 years (range 47–80 years). The median BMI was 28 kg/m^2^ (range 23–33 kg/m^2^). Ten patients (59%) had received neoadjuvant therapy. Cancer location was in the mid-rectum in 15 (88%) and in the low rectum in 2 (12%) patients. The register of operated patients and a summary of the patients’ characteristics are provided in Tables 1 and 2, respectively.

**Table 1:** Register of operated patients

Patient	Gender	Age	BMI	ASA class	Tumor distance from anal verge (cm)	LOS (days)	EBL (ml)	Time to transection (min)	Total operative time (min)	Anastomosis	Neoadjuvant therapy	No. of used trocars	Pathologic finding
1	F	73	24	III	8	8	20	179	330	DS S-E	CRT	6	ypT2, ypTN0 (0/14), R0, DRM: 3 cm
2	M	66	30	II	5	7	20	280	458	DS S-E	CRT	7	ypT2, ypN0 (0/15), R0, DRM: 1 cm
3	M	47	29	II	10	10	20	165	287	DS S-E	CRT	7	ypT2, ypN0 (0/12), R0, DRM: 6 cm
4	M	68	31	II	11	13	50	252	349	DS S-E	None	7	pT3c, pN1b (3/21), R0, DRM: 8 cm
5	F	66	25	II	8	8	20	115	217	DS S-E	CRT	6	ypT3b, ypN0 (0/14), R0, DRM: 5 cm
6	F	80	24	IV	10	12	40	133	270	Hartmann	None	6	pT3d, pN2a (6/22), R0, DRM: 5 cm
7	F	64	23	III	10	9	5	119	199	DS S-E	CRT	6	pCR, ypT0, ypN0 (0/14), R0, DRM: 2.5 cm
8	F	54	26	II	11	8	5	147	200	DS S-E	None	6	pT1, pN0 (0/16), R0, DRM: 6 cm
9	M	65	28	III	10	8	50	188	276	DS S-E	CRT	7	pCR, ypT0, ypN0 (0/14), R0, DRM: 3.5 cm
10	M	59	25	II	7	7	30	151	192	DS S-E	CRT	6	ypT2, ypN0 (0/20), R0, DRM: 1.5 cm
11	M	54	29	II	10	8	20	160	360	DS S-E	RT	7	ypT2, ypN2b (7/24), R0, DRM: 3 cm
12	M	72	30	III	8	14	40	137	352	Manual coloanal E-E	CRT	7	ypT2, ypN0 (0/12), R0, DRM: 2.2 cm
13	F	78	33	IV	12	11	50	152	260	DS S-E	None	7	pT2, pN0 (0/25), R0, DRM: 11 cm
14	M	60	33	II	8	8	40	155	285	DS S-E	None	7	pT1, pN0 (0/14), R0, DRM: 7 cm
15	F	65	30	II	5	12	30	134	272	DS S-E	None	6	pT3b, pN0 (0/19), R0, DRM: 1 cm
16	M	76	27	II	9	9	70	214	307	DS S-E	None	7	pT3c, pN2b (26/28), R0, DRM: 6 cm
17	M	71	25	IV	11	13	60	168	268	DS S-E	RT	7	ypT3b, ypN1b (2/12), R0, DRM: 5.6 cm

*BMI* body mass index, *ASA* American Society of Anesthesiologists, *LOS* length of stay, *EBL* estimated blood loss, *DRM* distal resection margin, *CRT* chemoradiotherapy, *DS* double-stapled, *S-E* side-to-end, *E-E* end-to-end

**Table 2:** Patient characteristics

Variable	Value
Median age (years)	66 (47–80)
Sex (F/M)	7/10
Median BMI (kg/m^2^)	28 (23–33)
ASA class	II (10), III (4), IV (3)
Rectal cancer location (middle/lower third)	Middle third (15), lower third (2)
Neoadjuvant therapy (nCRT, nRT or none)	nCRT (8), nRT (2), none (7)

*BMI* body mass index, *ASA* American Society of Anesthesiologists, *nCRT* chemoradiotherapy, *nRT* radiotherapy

#### Intraoperative and postoperative results

There were no intraoperative complications. All procedures were performed laparoscopically with the ARTISENTIAL^®^ monopolar spatula and the bipolar forceps. There were no conversions to laparotomy and no switch to using standard laparoscopic instruments. No additional vessel sealing devices had to be used. Sixteen patients received an anastomosis. A double-stapled side-to-end anastomosis was performed in 15 patients. A hand-sewn coloanal anastomosis was done in one patient. All anastomoses were located at or below 4 cm from the anal verge. A loop ileostomy was fashioned in all patients who received an anastomosis. One Hartmann procedure was performed in an American Society of Anesthesiologists (ASA) class 4, 80-year-old woman, on platelet aggregation inhibitor therapy, with evident intraoperative bowel distension in semi-stenotic disease (No. 6 in the register). She had had a myocardial infraction and coronary intervention 2 weeks before surgery.

The median time to rectal transection was 155 min (range 118–280 min). The median total operative time was 276 min (range 192–458 min). BMI (≥ 30 kg/m^2^) did not significantly affect the time to rectal transection (*p* = 0.6153, Kruskal–Wallis test) or the total operative time (*p* = 0.1317, Kruskal–Wallis test) in this study. The time to rectal transection and the total operative time were, however, significantly shorter in female patients (*p* = 0.0112 and 0.0404, respectively, Kruskal–Wallis test). The median EBL was 30 ml (range 5–70 ml). The median number of used trocars was 7 (range 6–7). In this study, men were significantly more likely to be operated on with 7 trocars than women (*p* = 0.0037, Fisher exact test). BMI (≥ 30 kg/m^2^), however, was not significantly associated with an increased number of trocars used (*p* = 0.3043, Fisher exact test).

**Fig. 5:**
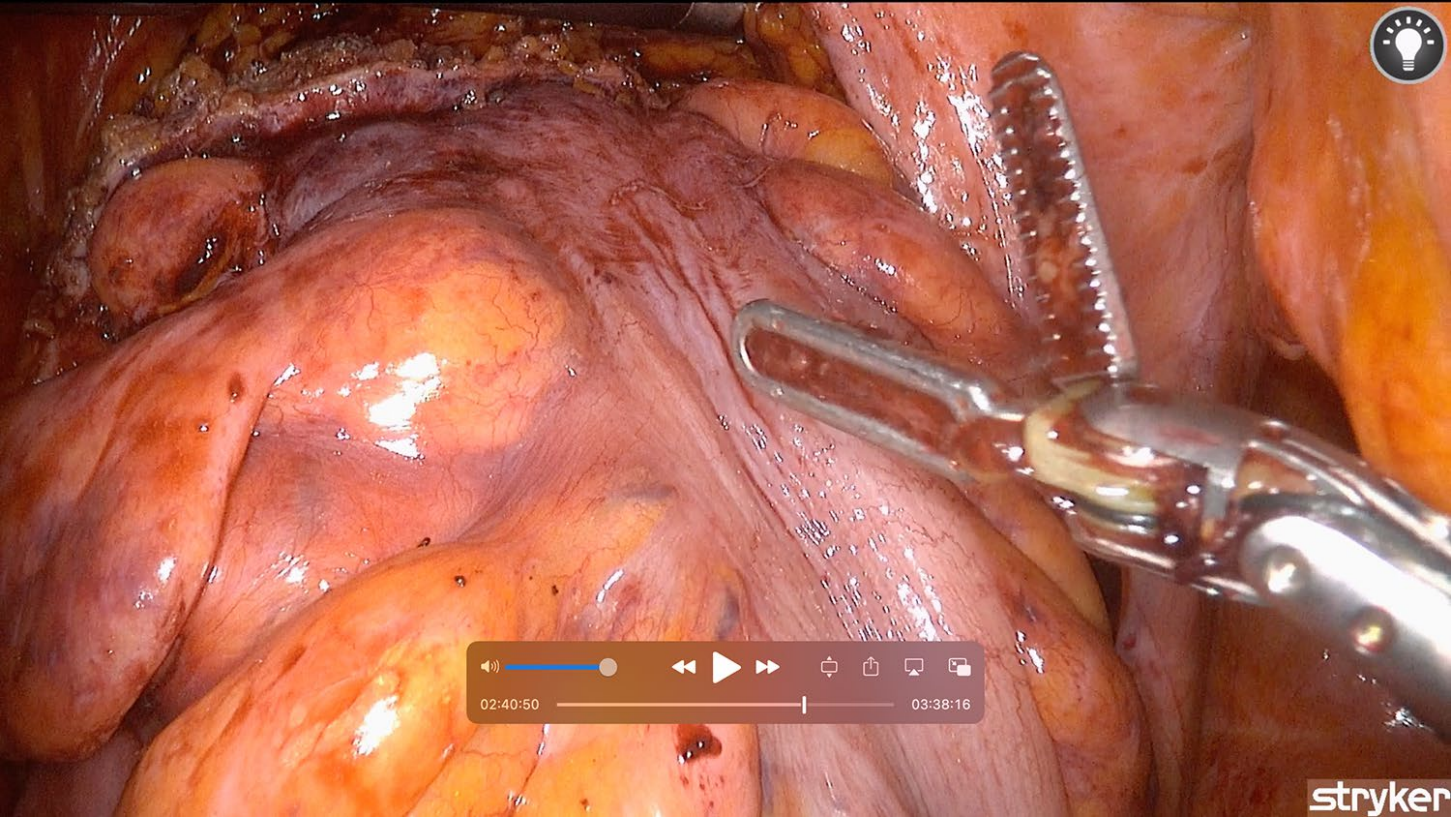
A picture showing the bent right jaw of the ARTISENTIAL^®^ forceps with jaw misalignment at the 160th minute of surgery

In one male patient (No. 16 in the register), the right jaw of the ARTISENTIAL^®^ forceps end effector bent at its base in the 160th minute of surgery while performing the TME, causing misalignment of the jaws (Fig. 5). This malfunction occurred while trying to push the mesorectum and the very bulky rectal tumor from the left to the right side using the forceps. The bent jaw was straightened out with a conventional laparoscopic grasper so as to enable safe removal through the trocar. The malfunctioned ARTISENTIAL^®^ forceps was replaced with a new instrument and surgery was continued.

The median length of stay was 9 days (range 7–14 days). The first bowel movement occurred on day 1 as a median (range 0–4 days). The intra- and postoperative results are summarized in Table 3.

**Table 3:** Intraoperative and postoperative results

Variable	Value or median value (range)
Time to transection (min)	155 (115–280)
Total operative time (min)	276 (192–458)
Number of trocars used	7 (6–7)
EBL (ml)	30 (5–70)
First bowel movement (DAS)	1 (0–4)
LOS (days)	9 (7–14)
Conversion	
CL	0
CSL	0
Instrument malfunction *n* (%)	1 (3%)

*EBL* estimated blood loss, *LOS* length of stay, *CL* conversion to laparotomy, *CSL* conversion to standard straight laparoscopic devices *DAS* days after surgery

#### Technical aspects

**Fig. 6:**
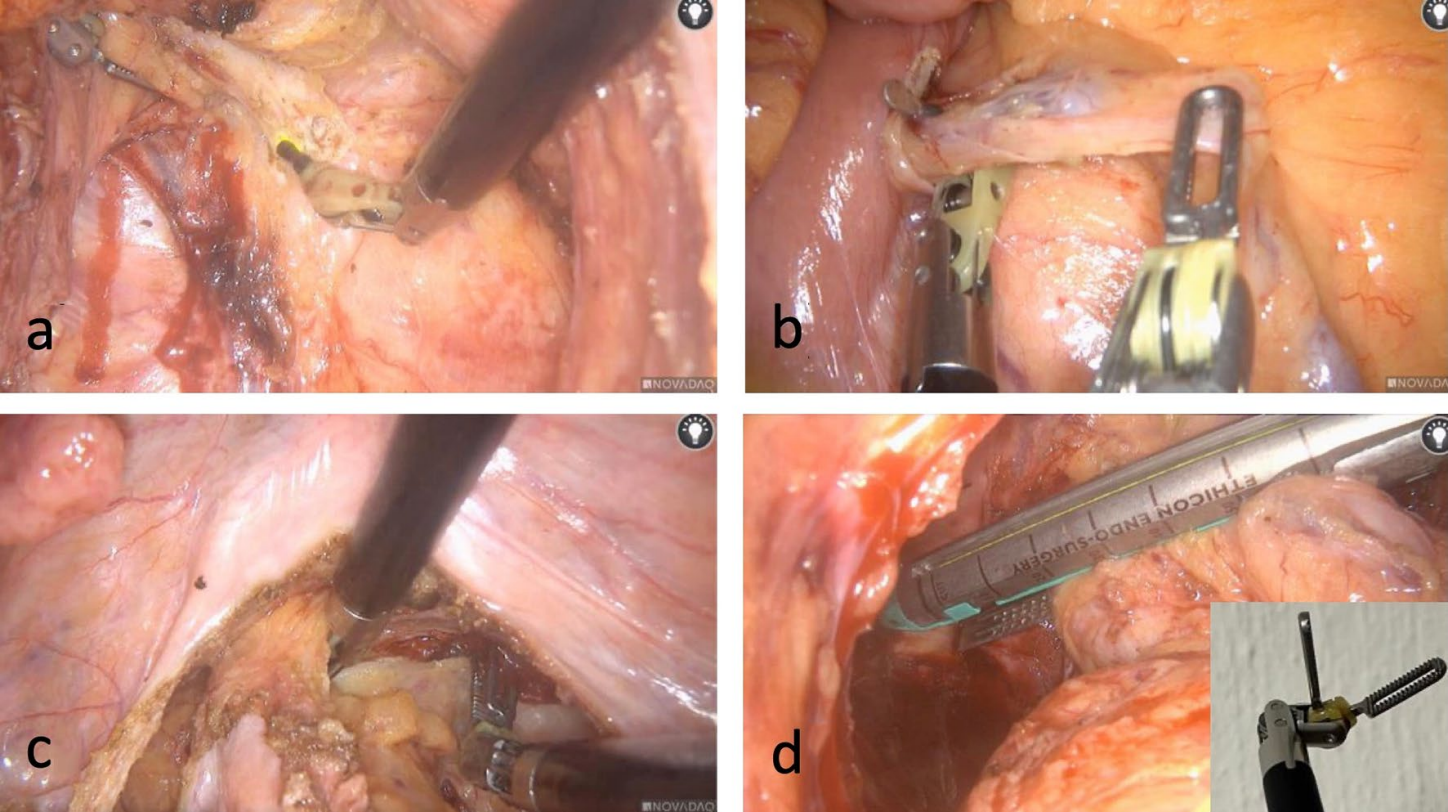
Intraoperative photos showing different technical advantages of articulation

The articulation provided by ARTISENTIAL^®^ appeared to be particularly advantageous in four specific circumstances among others (Video 1). (1) Dissection at the level of the rectococcygeal muscle which could be performed with relative ease due to a 100°-angle between the end effector and the shaft of the ARTISENTIAL^®^ spatula while lifting the mesorectum upwards with a 90° angled end effector of the ARTISENTIAL^®^ forceps (Fig. 6a); (2) easy preparation of the inferior mesenteric vessels (Fig. 6b); (3) performing swift hemostasis with the ARTISENTIAL^®^ bipolar forceps at any area in the pelvis, even those difficult to reach in the conventional laparoscopic and open technique (Fig. 6c); (4) assisting in transection of the rectum with a single firing by exploiting the advantage of double-jointed articulation of the ARTISENTIAL^®^ forceps to dorsally push the rectum into the branches of the endostapler (Fig. 6d).

#### Pathologic outcome

Pathologic assessment revealed good specimen quality with complete TME (Quirke Classification) in all patients [34–36]. R0 resection was confirmed in all patients with no cases of circumferential resection margin involvement. The median number of harvested lymph nodes was 15 (12–28). The median length of the distal resection margin was 5 cm (range 1–11 cm). The pathological findings in all patients are summarized in Table 4.

**Table 4:** Pathologic outcomes of all patients

Variable	Value
pT0, pN0: pCR, *n* (%)	2 (11.8%)
pT1, pN0, *n* (%)	2 (11.8%)
pT2, pN0, *n* (%)	6 (35.3%)
pT2, pN+, *n* (%)	1 (5.9%)
pT3, pN0, *n* (%)	2 (11.8%)
pT3, pN+, *n* (%)	4 (23.5%)
R0, *n* (%)	17 (100%)
Median number of lymph nodes, *n* (range)	15 (12–28)
DRM (cm), median (range)	5 (1–11)
CRM-negative	17 (100%)
Good TME quality (Quirke Classification), *n* (%)	17 (100%)

*pCR* pathologic complete response, *DRM* distal resection margin, *CRM* circumferential resection margin, *TME* total mesorectal excision

#### Complications

Fourteen patients (82.4%) had an uneventful postoperative course. Three patients (17.6%) had a Clavien–Dindo IIIb complication and had to be reoperated on.

A 68-year-old man (No. 4 in the register) developed postoperative small bowel ileus which failed to resolve with conservative treatment. Re-laparoscopy on postoperative day 4 revealed entrapment of a small bowel loop under the mesentery of the descending colon. The small bowel loop was released without complications. The patient had an uneventful postoperative course afterwards. He was discharged on day 13 after primary surgery (Table 5).

**Table 5:** Overall morbidity

Postoperative complications	*n* (%)
Characteristics	
Ileus	1 (5.9)
Bleeding	2 (11.8)
Anastomotic leak	0 (0)
Other	0 (0)
Readmission (unrelated)	1 (6)
Classification	
Clavien–Dindo IIIb	3 (17.6)
Mortality	0 (0)

One 80-year-old woman (No. 6 in the register), on platelet aggregation inhibitor therapy, had abdominal wall minor vessel bleeding at the drain site. Re-laparoscopy was done on postoperative day 1 and hemostasis was performed with no further complications. The patient had an uneventful postoperative course afterwards and was discharged on day 12 after primary surgery.

The third complication occurred in a 71-year-old man (No. 17 in the register) and involved abdominal wall ileostomy-site bleeding out of a branch of the inferior epigastric artery. Re-laparoscopy and hemostasis were performed a few hours after primary surgery with no further complications. The patient was discharged on day 13 after primary surgery.

There were no further complications. No symptomatic grade B and grade C anastomotic leaks were recorded in this study. Furthermore, at the time of writing, 15 patients have already had a reversal of their loop ileostomy after anastomotic integrity was evaluated with digital rectal examination, rectoscopy and a water-soluble contrast enema. Anastomotic leak was ruled out in the last patient still awaiting the reversal of his ileostomy.

There was no mortality in this study. One patient was readmitted nearly 1 week after discharge with biliary obstruction, gallbladder hydrops and cholecystitis.

#### Learning curve

**Fig. 7:**
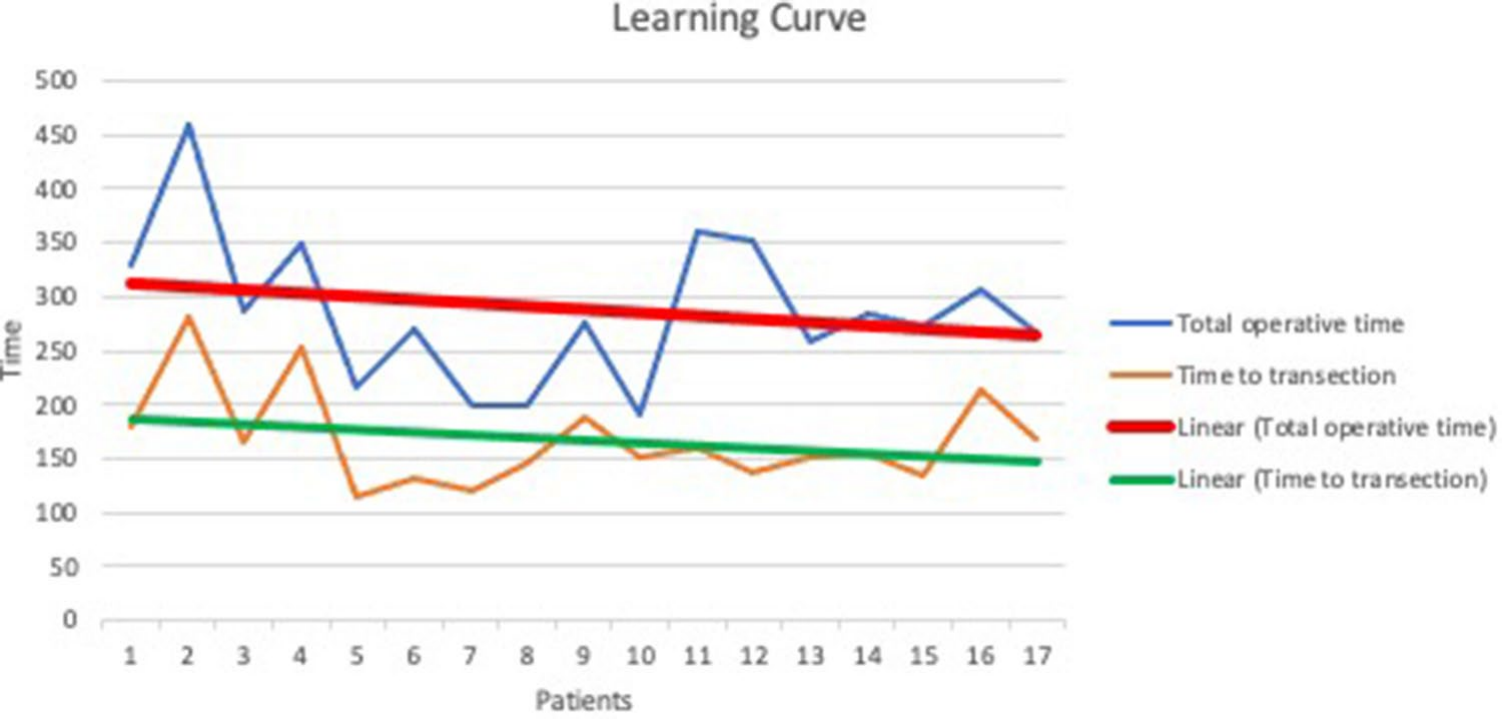
A graph showing the time to rectal transection and the total operative time in all performed procedures. The linear shows a trend of decreasing times

**Fig. 8:**
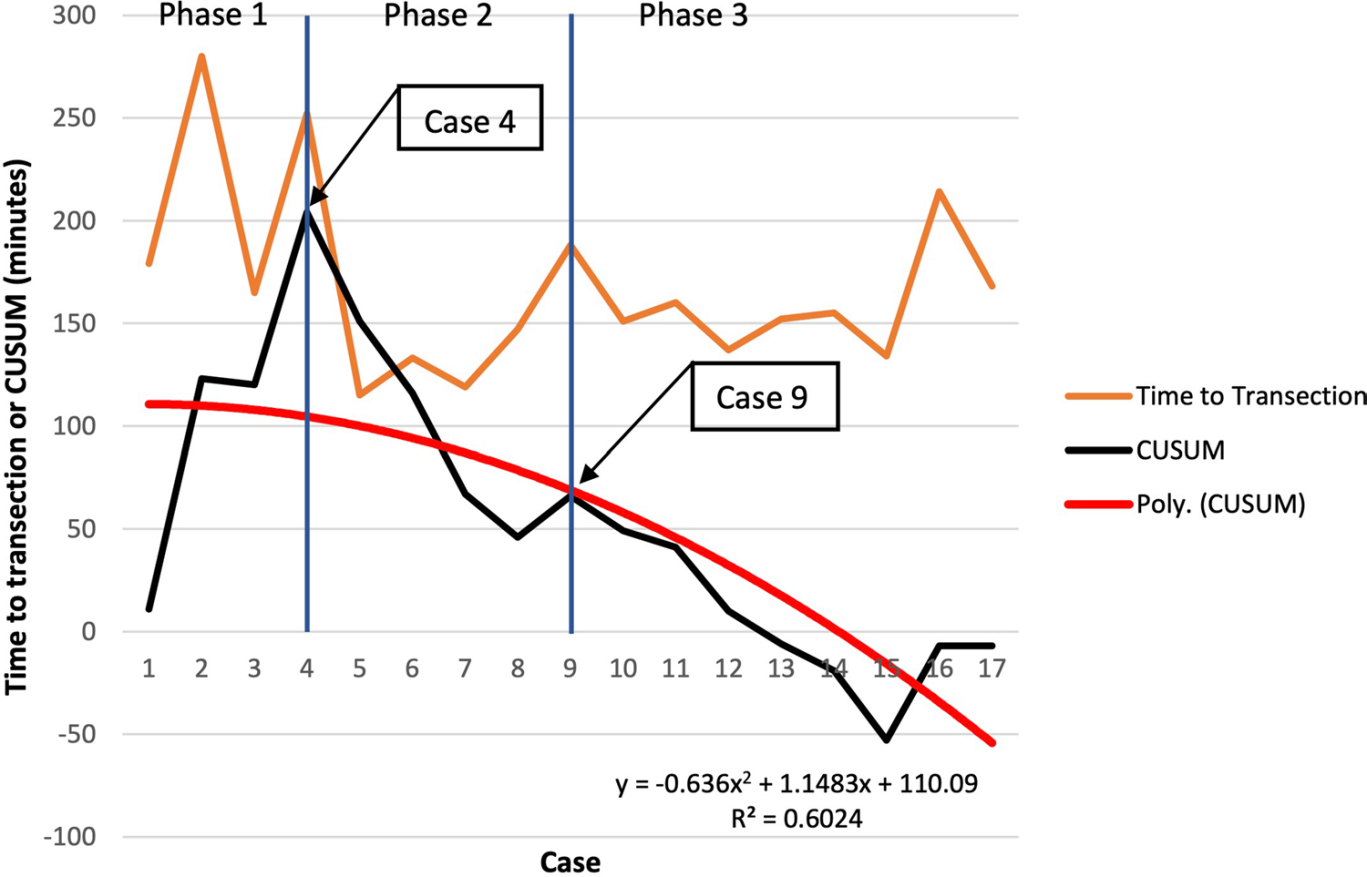
A graph showing the time to rectal transection and CUSUM in all performed procedures. The red curve shows a second-order polynomial with the formula − 0.636 × (case number)^2^ + 1.1483 × (case number) + 110.09, *R*^2^ = 0.6024. Three different CUSUM learning curve phases are identified

There was a trend towards decreasing times to rectal transection as well as decreasing total operative times in this study (Fig. 7). The CUSUM learning curve (Fig. 8) was plotted as a second-order polynomial with the formula − 0.636*x*^2^ + 1.1483*x* + 110.09 (*x* = case number) and *R*^2^ = 0.6024. Three CUSUM learning curve phases were identified: phase 1 (the first 4 cases), phase 2 (the next 5 cases) and phase 3 (the last 8 cases). The time to transection decreased significantly in phase 3 compared to phase 1 (*p* = 0.007) and phase 2 (*p* = 0.0012), respectively. There was no significant decrease in the time to transection between phase 1 and phase 2 (*p* = 0.5551).

### Discussion

Case reports and video vignettes that reported feasibility and safety of the use of ARTISENTIAL^®^ in colorectal surgery have already been published [18, 22, 23]. This study now describes the first experience of using ARTISENTIAL^®^ in a larger cohort of patients undergoing a laparoscopic LAR with TME. All procedures in this study were performed entirely with ARTISENTIAL^®^ with no cases of conversion to laparotomy or switching to standard laparoscopic instruments. The complication rate was 17.6%. However, the complications in this study were unrelated to the use of ARTISENTIAL^®^. There were no mortalities and only one unrelated readmission. The pathologic or oncologic outcome was adequate. No anastomotic leaks were recorded.

#### Conversion rate and operative times

No conversions to laparotomy were recorded despite a male majority and a relatively high BMI in this prospective study, without patient selection. This might suggest a benefit of using articulated devices in terms of better accessibility in confined anatomical spaces. This compares well with data that suggest lower conversion-to-open rates with robotic assistance in comparison to standard laparoscopy [8, 37].

In the literature, BMI has frequently been identified as an independent predictor of prolonged operative times in laparoscopic colorectal surgery [38, 39]. In this study, BMI (≥ 30 kg/m^2^) did not significantly affect the time to rectal transection or the total operative time. One might consider that articulation played a role in improving the operative times. However, the small number of patients and the fact that most of the obese patients in this study were males (median BMI: males 29 kg/m^2^, females 25 kg/m^2^) may have been more relevant in contributing to this result. Indeed, time to rectal transection and the total operative time were significantly longer in males in this study (*p* = 0.0112 and 0.0404, respectively). This is in line with evidence from the literature noting longer operative times in laparoscopic colorectal resections in males [40]. In a similar manner, male sex was significantly associated with placing an additional ancillary port in this study. This is also in line with the literature showing increased surgical difficulty in the narrow male pelvis [41, 42].

Learning curve analysis showed a trend towards decreasing operative times in general. Furthermore, CUSUM method analysis revealed a significant decrease in the time to rectal transection after the ninth procedure, suggesting at least an improved competency in using the ARTISENTIAL^®^ instruments beyond that point.

#### Complications

There were no intraoperative complications in this study. This coincides with data coming from robotic assisted colorectal resections where intraoperative complications as low as 1.5% were reported [43]. However, Jayne et al. reported intraoperative complication rates of ca. 15% in both laparoscopic and robotic assisted low anterior resection [12]. The median EBL of 30 ml (range 5–70 ml) was in general very low in our study. This compares well with data from the literature showing minimal estimated blood loss in robotic assisted LAR in comparison to standard laparoscopy [44].

The 30-day postoperative complication rate in this study was 17.6%. All three complications were rated Clavien–Dindo IIIb since the patients had to be reoperated on. This also coincides with evidence from the literature which defines rectal cancer surgery as a high-risk intervention [12]. However, it should be noted that the three complications were deemed to be unrelated to the use of ARTISENTIAL^®^. There were two cases of abdominal wall bleeding that occurred at the site of the drain and the loop ileostomy site, respectively. The third complication was an ileus that resulted from entrapment of the small bowel under the mesentery of the descending colon.

The absence of intraoperative complications in this study combined with a minimal EBL might have been influenced by the small number of patients. Yet, this may also suggest that articulation, combined with the familiar haptic of laparoscopy, might have enhanced surgical precision and prevented unintended iatrogenic injuries.

No symptomatic anastomotic leaks were recorded in this study. On follow-up, reversal of the loop ileostomy had already been performed in 15 out of 16 patients with an anastomosis at the time this paper was written. Anastomotic leak was also ruled out in the last patient still awaiting stoma reversal. In all of these patients, anastomotic integrity was evaluated with digital rectal examination, rectoscopy and a water-soluble contrast enema, thus ruling out a grade A anastomotic leak. This result may have also been influenced by the small number of patients in this study. Yet, one must consider that all procedures were performed by two high-volume and expert colorectal surgeons. All patients received mechanical bowel prep with oral antibiotics 1 day before surgery. A high-tie of the inferior mesenteric artery and taking down of the splenic flexure was performed in all patients. Furthermore, bowel perfusion was assessed with indocyanine green-enhanced fluorescence angiography in all cases.

#### Pathologic outcome

The immediate oncologic outcome in this series was adequate with good TME quality, satisfactory number of harvested lymph nodes and no circumferential margin involvement. This result suggests that the use of ARTISENTIAL^®^ in performing proper oncologic rectal surgery is feasible and safe.

#### Length of stay

The median length of stay in this series was 9 days (range 7–14 days). This relatively long length of stay can be partly explained by the previously described complications. Yet, other factors, specifically related to German health insurance payment methods and coordination of post-inpatient care and rehabilitation, played a major role in this study in dictating the length of stay, so that this criterium might not be representative for the clinical outcome. According to a EUROSTAT’s survey from 2016, Germany ranked fourth among European countries for the longest average inpatient stay in general.

#### *ARTISENTIAL*^*®*^* performance*

On a technical note, the ARTISENTIAL^®^ instruments proved to be helpful in performing otherwise difficult tasks in confined anatomical spaces (Fig. 6). One of the most striking features was the ability to apply swift hemostasis at any angle with the bipolar forceps in extremely narrow spaces in the pelvis (Video 1). Considering the fact that these single-use articulated instruments are readily available under any circumstances, advantages in everyday surgical practice, especially in the emergency setting, can be anticipated. Furthermore, combining the use of these instruments with 3D-laparoscopy could prove to be an affordable alternative to expensive robotic platforms. Obviously, studies are needed to examine these possibilities.

Furthermore, ARTISENTIAL^®^ proved in this study to be durable. With the exception of one malfunction with a bent jaw of the bipolar forceps (1 out of 34 used instruments: 3%), no other malfunctions or instrument failures were recorded despite relatively long usage times. More frequent malfunctions have been described in standard laparoscopic instruments and in surgical robotic systems that have been attributed to instrument mishandling and aggressive intraoperative use [45–47]. In our case, the male patient had a very bulky and large tumor which may have contributed to a higher mechanical burden on the device.

#### Study limitations

This study has several limitations. First, the small number of patients may have fallen short of being able to reflect similar results to those invariably described in the literature, like in the case of anastomotic leaks which did not occur in this study or the operative times which did not seem to be significantly affected by high BMI. Second, the study analysis was retrospective despite the prospective nature of patient enrollment and data collection. Third, this cohort study does not compare ARTISENTIAL^®^ to standard laparoscopic instruments or to robotic assisted surgery due to its descriptive nature. This paves the way, however, for a larger comparative study that evaluates the benefits of ARTISENTIAL^®^.

## Conclusions

This study shows that ARTISENTIAL^®^ assisted surgery can be safe and feasible in performing a laparoscopic low anterior resection with TME with adequate clinical and short-term oncologic outcome. Comparative and randomized trials are needed to elaborate more on the results of this work.

## Supplementary Information

Below is the link to the electronic supplementary material.

Supplementary file1 (MP4 295307 KB)
